# Supplementary material for: Sex classification from functional brain connectivity: Generalization to multiple datasets
Source: Hum Brain Mapp. 2024 Apr 22;45(6):e26683. doi: 10.1002/hbm.26683 (PMC11034006; doi:10.1002/hbm.26683)
Supplement: Supplementary file 1 — DATA S1: Supporting Information. [file HBM-45-e26683-s001.docx]

Supplementary Materials for

**Sex classification from functional brain connectivity: Generalization to multiple datasets**

Lisa Wiersch^1,2^, Patrick Friedrich^1,2^, Sami Hamdan^1,2^, Vera Komeyer^1,2,3^, Felix Hoffstadter^1,2^, Kaustubh R. Patil^1,2^, Simon B. Eickhoff^1,2^ and Susanne Weis^1,2^

^1^Institute of Systems Neuroscience, Heinrich Heine University Düsseldorf, Düsseldorf, Germany

^2^Institute of Neuroscience and Medicine (INM-7: Brain and Behaviour), Research Centre Jülich, Jülich, Germany

^3^Department of Biology, Faculty of Mathematics and Natural Sciences, Heinrich Heine University Düsseldorf, Düsseldorf, Germany

This file includes:

Figure S1 to S3

Table S1 to S3

**Supplementary Results**

**
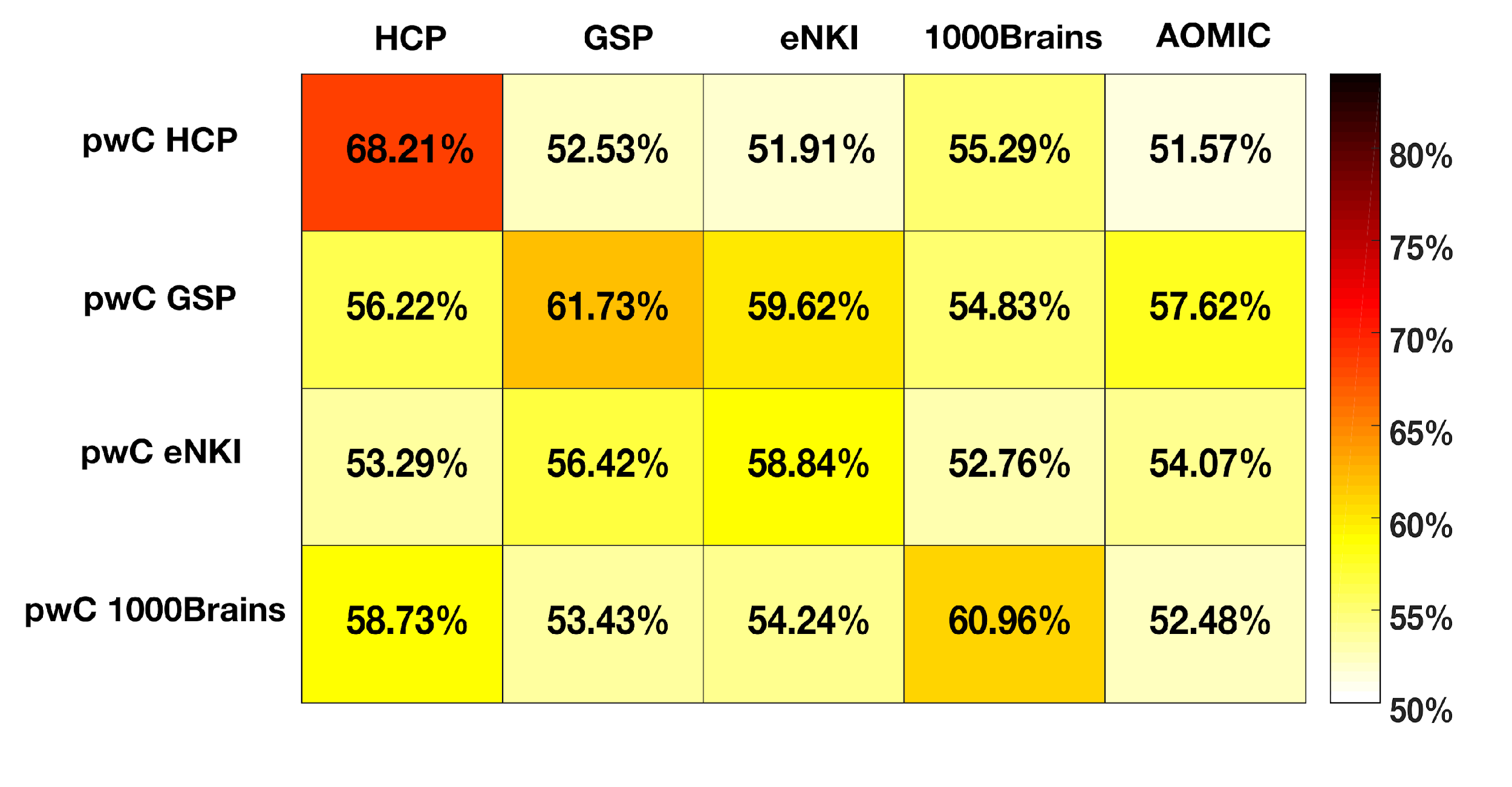
**

**Figure S1. Tile Plots of mean accuracies.** Mean accuracies averaged across all 436 parcels for each CV- and across-sample predictions of pwCs trained on the data of single samples.

**
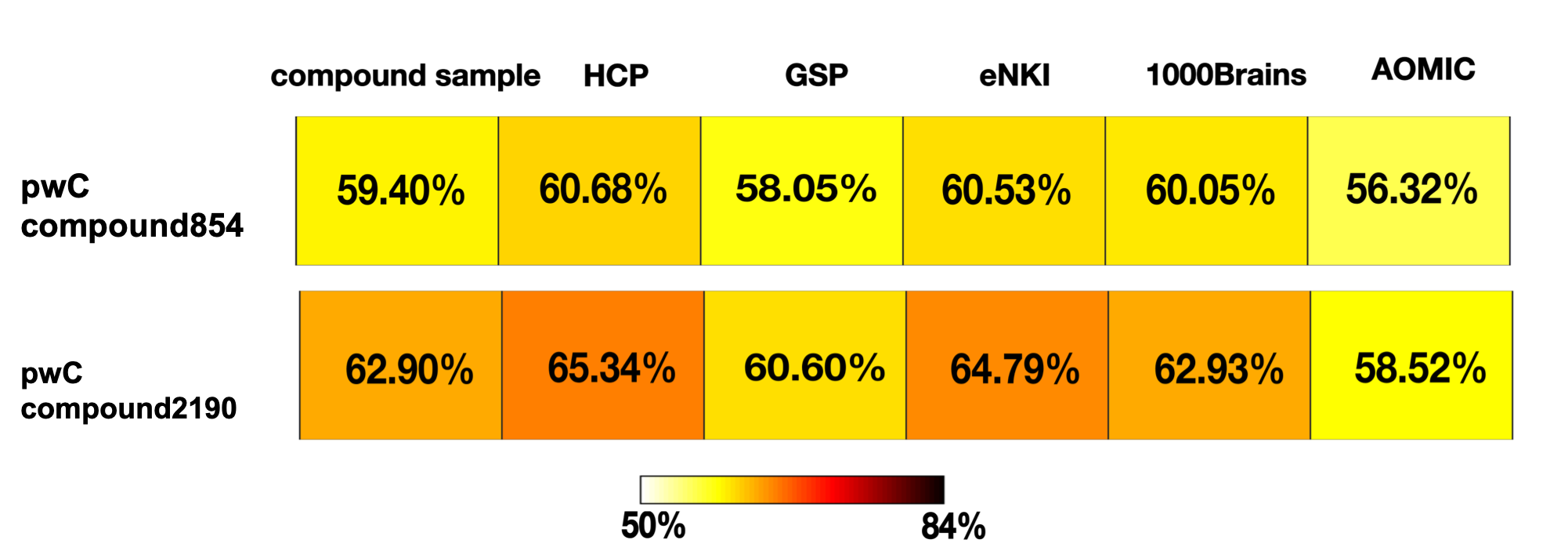
**

**Figure S2. Tile Plots of mean accuracies.** Mean accuracies averaged across all 436 parcels for the CV- and across-sample predictions of pwC compound854 and pwC compound2190.

**
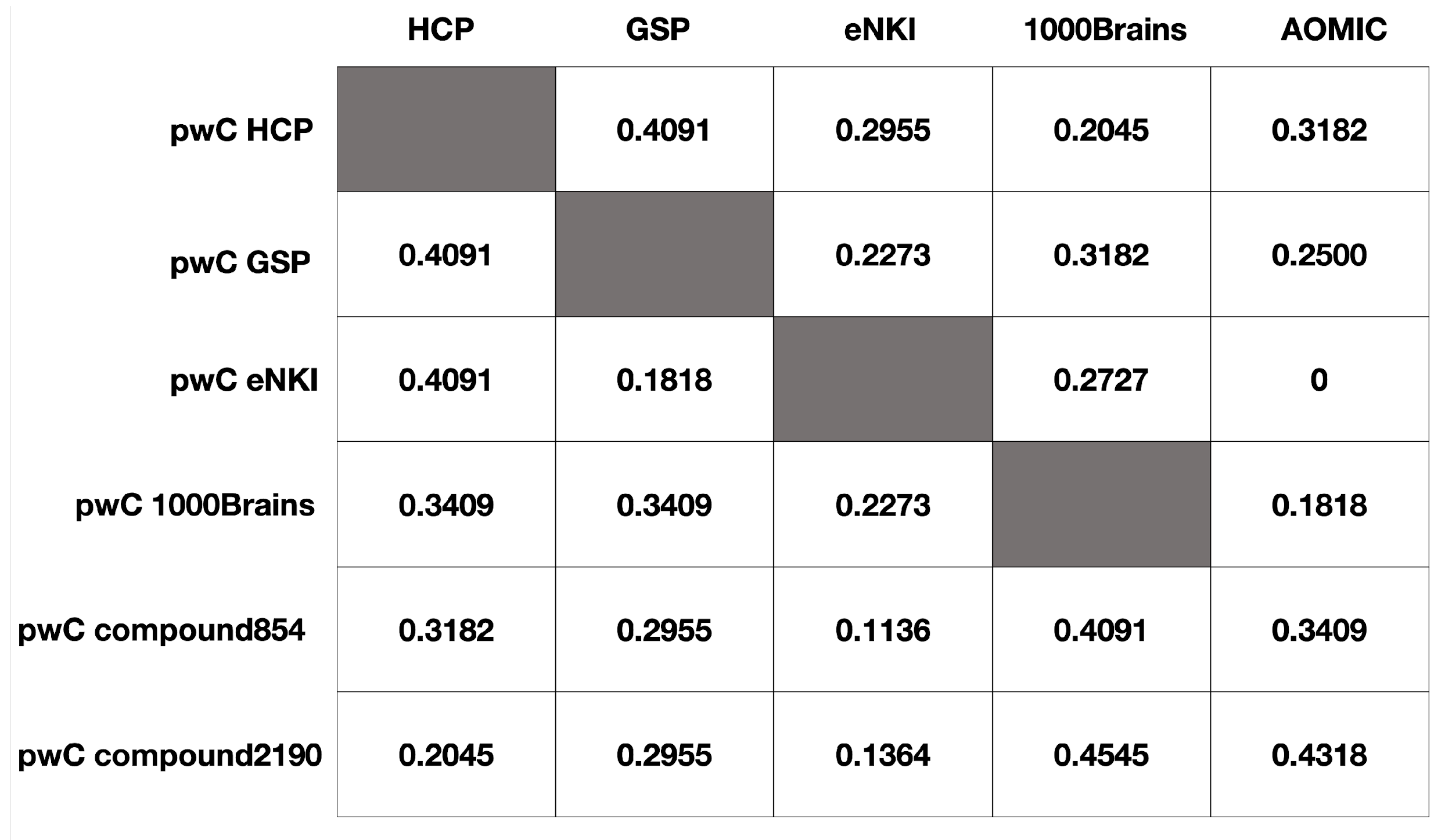
**

**Figure S3. Spatial consistency of all pwCs.** For each combination of training (rows) and test sample (columns), dice coefficients were calculated as the number of similar parcels classifying within the top 10% parcels for each CV- and across-sample prediction

| **Table S1** |  |  |  |  |  |  |  |  |  |  |  |  |
| --- | --- | --- | --- | --- | --- | --- | --- | --- | --- | --- | --- | --- |
| Mean and range in percent of sex classification accuracies for within- and between-dataset predictions | | | | | | | | | | |  |  |
|  |  |  |  |  |  |  |  |  |  |  |  |  |
|  |  |  |  | **Model applied to** | | | | | | |  |  |
|  |  | **compound sample (CV-prediction)** |  | **HCP** |  | **GSP** |  | **eNKI** |  | **1000Brains** |  | **AOMIC** |
|  |  |  |  |  |  |  |  |  |  |  |  |  |
| **pwC HCP** |  |  |  | 68.2 (51.0 - 77.4) |  | 52.5 (49.0 - 64.0) |  | 51.9 (44.7 - 65.3) |  | 55.3 (49.0 - 61.8) |  | 51.6 (47.1 - 65.0) |
|  |  |  |  |  |  |  |  |  |  |  |  |  |
| **pwC GSP** |  |  |  | 56.2 (46.0 - 67.3) |  | 61.7 (52.4 - 68.5) |  | 59.6 (49.0 - 70.0) |  | 54.8 (48.1 - 64.1) |  | 57.6 (50.0 - 68.1) |
|  |  |  |  |  |  |  |  |  |  |  |  |  |
| **pwC eNKI** |  |  |  | 53.3 (46.0 - 63.9) |  | 56.4 (48.7 - 63.8) |  | 58.8 (47.3 - 72.1) |  | 52.8 (45.8 - 62.5) |  | 54.1 (45.2 - 65.0) |
|  |  |  |  |  |  |  |  |  |  |  |  |  |
| **pwC 1000Brains** |  |  |  | 58.7 (49.4 - 68.7) |  | 53.4 (49.5 - 65.2) |  | 54.2 (47.4 - 66.8) |  | 61.0 (54.2 - 69.3) |  | 52.5 (46.5 - 66.2) |
|  |  |  |  |  |  |  |  |  |  |  |  |  |
| **pwC compound854** |  | 59.4 (50.8 - 67.5) |  | 60.7 (45.9 - 75.0) |  | 58.1 (46.3 - 70.0) |  | 60.5 (41.7 - 81.3) |  | 60.1 (44.4 - 72.0) |  | 56.3 (46.2 - 67.2) |
|  |  |  |  |  |  |  |  |  |  |  |  |  |
| **pwC compound2190** |  | 62.9 (53.7 - 70.1) |  | 65.3 (45.9 - 74.6) |  | 60.6 (49.1 - 70.1) |  | 64.8 (47.9 - 83.3) |  | 62.9 (52.4 - 75.2) |  | 58.5 (49.3 - 69.0) |

**Table S2.** Comparisons of performance of the 10% best classifying parcels each pwC on the different test samples (a) and comparisons of performance of the different pwCs on each test samples (b)

(a)

| **pwC HCP** |  |  |  |  |
| --- | --- | --- | --- | --- |
|  | **AOMIC** | **1000Brains** | **eNKI** |  |
| **GSP** | t = 1.40, p = 0.1643 | t = -2.23, p = 0.0281 | t = 1.17, p = 0.2464 |  |
| **eNKI** | t = 0.33, p = 0.7387 | t = -3.58, p = 0.005 |  |  |
| **1000Brains** | t = 3.50, p = 0.007 |  |  |  |
|  |  |  |  |  |
|  |  |  |  |  |
| **pwC GSP** |  |  |  |  |
|  | **AOMIC** | **1000Brains** | **eNKI** |  |
| **HCP** | t = 1.87, p = 0.0649 | t = 9.97, p < 0.0001 | t = -7.79, p < 0.0001 |  |
| **eNKI** | t = 9.27, p < 0.0001 | t = 16.81, p < 0.0001 |  |  |
| **1000Brains** | t = -7.83, p < 0.0001 |  |  |  |
|  |  |  |  |  |
|  |  |  |  |  |
| **pwC eNKI** |  |  |  |  |
|  | **AOMIC** | **1000Brains** | **GSP** |  |
| **HCP** | t = 2.88, p = 0.0050 | t = 5.48, p < 0.0001 | t = -1.83, p = 0.0711 |  |
| **GSP** | t = 5.66, p < 0.0001 | t = 8.76, p < 0.0001 |  |  |
| **1000Brains** | t = -2.96, p = 0.0039 |  |  |  |
|  |  |  |  |  |
|  |  |  |  |  |
| **pwC 1000Brains** |  |  |  |  |
|  | **AOMIC** | **eNKI** | **GSP** |  |
| **HCP** | t = 13.02, p < 0.0001 | t = 6.72, p < 0.0001 | t = 14.03, p < 0.0001 |  |
| **GSP** | t = 1.67, p = 0.0992 | t = -5.85, p < 0.0001 |  |  |
| **eNKI** | t = 6.56, p < 0.0001 |  |  |  |
|  |  |  |  |  |
|  | alpha level: 0.008 |  |  |  |
|  |  |  |  |  |
| **pwC compound854** | |  |  |  |
|  | **AOMIC** | **1000Brains** | **eNKI** | **GSP** |
| **HCP** | t = 17.14, p < 0.0001 | t = 2.66, p = 0.0093 | t = -6.60, p < 0.0001 | t = 9.63, p < 0.0001 |
| **GSP** | t = 8.38, p < 0.0001 | t = -7.74, p < 0.0001 | t = -15.79, p < 0.0001 | |
| **eNKI** | t = 22.55, p < 0.0001 | t = 9.46, p < 0.0001 |  |  |
| **1000Brains** | t = 16.29, p < 0.0001 |  |  |  |
|  |  |  |  |  |
|  | alpha level: 0.005 |  |  |  |
|  |  |  |  |  |
| **pwC compound2190** | |  |  |  |
|  | **AOMIC** | **1000Brains** | **eNKI** | **GSP** |
| **HCP** | t = 21.05, p < 0.0001 | t = 6.55, p < 0.0001 | t = -6.11, p < 0.0001 | t = 16.74, p < 0.0001 |
| **GSP** | t = 5.25, p < 0.0001 | t = -8.64, p < 0.0001 | t = -17.29, p < 0.0001 | |
| **eNKI** | t = 20.42, p < 0.0001 | t = 10.46, p < 0.0001 |  |  |
| **1000Brains** | t = 13.03, p < 0.0001 |  |  |  |
|  |  |  |  |  |
|  | alpha level: 0.005 |  |  |  |

|  |  |  |  |  |
| --- | --- | --- | --- | --- |

**(b)**

| **test sample HCP** |  |  |  |  |
| --- | --- | --- | --- | --- |
|  | **pwC compound854** | **pwC compound2190** | **pwC 1000Brains** | **pwC eNKI** |
| **pwC GSP** | t = -13.77, p < 0.0001 | t = -29.57, p < 0.0001 | t = -4.54, p < 0.0001 | t = 11.23, p < 0.0001 |
| **pwC eNKI** | t = -21.31, p < 0.0001 | t = -36.28, p < 0.0001 | t = -15.18, p < 0.0001 |  |
| **pwC 1000Brains** | t = -10.59, p < 0.0001 | t = -25.64, p < 0.0001 |  |  |
| **pwC compound2190** | t = 8.75, p < 0.0001 |  |  |  |
|  |  |  |  |  |
|  |  |  |  |  |
| **test sample GSP** |  |  |  |  |
|  | **pwC compound854** | **pwC compound2190** | **pwC 1000Brains** | **pwC eNKI** |
| **pwC HCP** | t = -15.44, p < 0.0001 | t = -22.16, p < 0.0001 | t = -3.68, p = 0.0004 | t = -4.84, p < 0.0001 |
| **pwC eNKI** | t = -14.89, p < 0.0001 | t = -24.64, p < 0.0001 | t = 0.71, p = 0.4816 |  |
| **pwC 1000Brains** | t = -12.93, p < 0.0001 | t = -20.43, p < 0.0001 |  |  |
| **pwC compound2190** | t = 7.01, p < 0.0001 |  |  |  |
|  |  |  |  |  |
|  |  |  |  |  |
| **test sample eNKI** |  |  |  |  |
|  | **pwC compound854** | **pwC compound2190** | **pwC 1000Brains** | **pwC GSP** |
| **pwC HCP** | t = -26.98, p < 0.0001 | t = -31.93, p < 0.0001 | t = -9.77, p < 0.0001 | t = -19.01, p < 0.0001 |
| **pwC GSP** | t = -14.07, p < 0.0001 | t = -19.97, p < 0.0001 | t = 9.33, p < 0.0001 |  |
| **pwC 1000Brains** | t = -19.97, p < 0.0001 | t = -25.36, p < 0.0001 |  |  |
| **pwC compound2190** | t = 5.11, p < 0.0001 |  |  |  |
|  |  |  |  |  |
|  |  |  |  |  |
| **test sample 1000Brains** | |  |  |  |
|  | **pwC compound854** | **pwC compound2190** | **pwC eNKI** | **pwC GSP** |
| **pwC HCP** | t = -27.12, p < 0.0001 | t = -35.92, p < 0.0001 | t = 5.66, p < 0.0001 | t = -4.22, p < 0.0001 |
| **pwC GSP** | t = -20.35, p < 0.0001 | t = -27.70, p < 0.0001 | t = 8.25, p < 0.0001 |  |
| **pwC eNKI** | t = -27.78, p < 0.0001 | t = -35.29, p < 0.0001 |  |  |
| **pwC compound2190** | t = 6.32, p < 0.0001 |  |  |  |
|  |  |  |  |  |
|  | alpha level: 0.005 |  |  |  |

| **test sample AOMIC** | |  |  |  |  |
| --- | --- | --- | --- | --- | --- |
|  | **pwC compound854** | **pwC compound2190** | **pwC 1000Brains** | **pwC eNKI** | **pwC GSP** |
| **pwC HCP** | t = -9.44, p < 0.0001 | t = -16.08, p < 0.0001 | t = -2.75, p = 0.0072 | t = -2.01, p = 0.0476 | t = -11.02, p < 0.0001 |
| **pwC GSP** | t = 2.66, p = 0.0094 | t = -7.78, p < 0.0001 | t = 8.41, p < 0.0001 | t = 13.60, p < 0.0001 | |
| **pwC eNKI** | t = -11.37, p < 0.0001 | t = -20.71, p < 0.0001 | t = -1.37, p = 0.1745 | |  |
| **pwC 1000Brains** | t = -6.66, p < 0.0001 | t = -13.92, p < 0.0001 | |  |  |
| **pwC compound2190** | t = 10.51, p < 0.0001 | |  |  |  |
|  |  |  |  |  |  |
|  | alpha level: 0.003 | |  |  |  |

**Table S3.** Comparisons of performance of all 436 parcels each pwC on the different test samples (a) and comparisons of performance of the different pwCs on each test samples (b)

**(a)**

| **pwC HCP** |  |  |  |  |
| --- | --- | --- | --- | --- |
|  | **AOMIC** | **1000Brains** | **eNKI** |  |
| **GSP** | t = 5.15, p < 0.0001 | t = -15.54, p < 0.0001 | t = 3.24, p = 0.0012 |  |
| **eNKI** | t = 1.84, p = 0.0654 | t = -19.01, p < 0.0001 |  |  |
| **1000Brains** | t = 21.45, p < 0.0001 |  |  |  |
|  |  |  |  |  |
|  |  |  |  |  |
| **pwC GSP** |  |  |  |  |
|  | **AOMIC** | **1000Brains** | **eNKI** |  |
| **HCP** | t = -5.69, p < 0.0001 | t = 5.64, p < 0.0001 | t = -13.26, p < 0.0001 | |
| **eNKI** | t = 8.89, p < 0.0001 | t = 21.35, p < 0.0001 |  |  |
| **1000Brains** | t = -13.13, p < 0.0001 |  |  |  |
|  |  |  |  |  |
|  |  |  |  |  |
| **pwC eNKI** |  |  |  |  |
|  | **AOMIC** | **1000Brains** | **GSP** |  |
| **HCP** | t = -3.72, p = 0.0002 | t = 2.55, p = 0.0108 | t = -15.86, p < 0.0001 | |
| **GSP** | t = 12.84, p < 0.0001 | t = 20.25, p < 0.0001 |  |  |
| **1000Brains** | t = -6.76, p < 0.0001 |  |  |  |
|  |  |  |  |  |
|  |  |  |  |  |
| **pwC 1000Brains** |  |  |  |  |
|  | **AOMIC** | **eNKI** | **GSP** |  |
| **HCP** | t = 27.54, p < 0.0001 | t = 17.76, p < 0.0001 | t = 23.19, p < 0.0001 |  |
| **GSP** | t = 4.43, p < 0.0001 | t = -3.35, p = 0.0008 |  |  |
| **eNKI** | t = 7.32, p < 0.0001 |  |  |  |
|  |  |  |  |  |
|  | alpha level: 0.008 |  |  |  |
|  |  |  |  |  |
|  |  |  |  |  |
| **pwC compound854** | |  |  |  |
|  | **AOMIC** | **1000Brains** | **eNKI** | **GSP** |
| **HCP** | t = 15.61, p < 0.0001 | t = 2.03, p = 0.0427 | t = 0.39, p = 0.6965 | t = 8.92, p < 0.0001 |
| **GSP** | t = 6.81, p < 0.0001 | t = -6.92, p < 0.0001 | t = -6.74, p < 0.0001 |  |
| **eNKI** | t = 11.83, p < 0.0001 | t = 1.26, p = 0.2073 |  |  |
| **1000Brains** | t = 13.66, p < 0.0001 |  |  |  |
|  |  |  |  |  |
|  | alpha level: 0.005 |  |  |  |
|  |  |  |  |  |
|  |  |  |  |  |
| **pwC compound2190** | |  |  |  |
|  | **AOMIC** | **1000Brains** | **eNKI** | **GSP** |
| **HCP** | t = 25.43, p < 0.0001 | t = 8.60, p < 0.0001 | t = 1.55, p = 0.1207 | t = 17.34, p < 0.0001 |
| **GSP** | t = 8.17, p < 0.0001 | t = -8.74, p < 0.0001 | t = -12.21, p < 0.0001 | |
| **eNKI** | t = 18.49, p < 0.0001 | t = 5.34, p < 0.0001 |  |  |
| **1000Brains** | t = 16.88, p < 0.0001 |  |  |  |
|  |  |  |  |  |
|  | alpha level: 0.005 |  |  |  |

**(b)**

|  |  |  |  |  |
| --- | --- | --- | --- | --- |
| **test sample HCP** |  |  |  |  |
|  | **pwC compound854** | **pwC compound2190** | **pwC 1000Brains** | **pwC eNKI** |
| **pwC GSP** | t = -15.04, p < 0.0001 | t = -32.42, p < 0.0001 | t = -9.73, p < 0.0001 | t = 11.71, p < 0.0001 |
| **pwC eNKI** | t = -27.06, p < 0.0001 | t = -47.01, p < 0.0001 | t = -23.56, p < 0.0001 | |
| **pwC 1000Brains** | t = -6.94, p < 0.0001 | t = -24.97, p < 0.0001 | |  |
| **pwC compound2190** | t = 15.41, p < 0.0001 |  |  |  |
|  |  |  |  |  |
|  |  |  |  |  |
| **test sample GSP** |  |  |  |  |
|  | **pwC compound854** | **pwC compound2190** | **pwC 1000Brains** | **pwC eNKI** |
| **pwC HCP** | t = -23.61, p < 0.0001 | t = -35.45, p < 0.0001 | t = -4.45, p < 0.0001 | t = -21.58, p < 0.0001 |
| **pwC eNKI** | t = -7.24, p < 0.0001 | t = -19.06, p < 0.0001 | t = 15.36, p < 0.0001 | |
| **pwC 1000Brains** | t = -18.85, p < 0.0001 | t = -29.98, p < 0.0001 | |  |
| **pwC compound2190** | t = 9.59, p < 0.0001 |  |  |  |
|  |  |  |  |  |
|  |  |  |  |  |
| **test sample eNKI** |  |  |  |  |
|  | **pwC compound854** | **pwC compound2190** | **pwC 1000Brains** | **pwC GSP** |
| **pwC HCP** | t = -25.23, p < 0.0001 | t = -40.26, p < 0.0001 | t = -10.12, p < 0.0001 | t = -35.92, p < 0.0001 |
| **pwC GSP** | t = -2.56, p = 0.0108 | t = -15.43, p < 0.0001 | t = 21.42, p < 0.0001 | |
| **pwC 1000Brains** | t = -17.20, p < 0.0001 | t = -30.54, p < 0.0001 | |  |
| **pwC compound2190** | t = 9.96, p < 0.0001 |  |  |  |
|  |  |  |  |  |
|  |  |  |  |  |
| **test sample 1000Brains** | |  |  |  |
|  | **pwC compound854** | **pwC compound2190** | **pwC eNKI** | **pwC GSP** |
| **pwC HCP** | t = -19.39, p < 0.0001 | t = -33.80, p < 0.0001 | t = 14.19, p < 0.0001 | t = 2.43, p = 0.0153 |
| **pwC GSP** | t = -19.86, p < 0.0001 | t = -33.09, p < 0.0001 | t = 10.27, p < 0.0001 | |
| **pwC eNKI** | t = -28.62, p < 0.0001 | t = -43.07, p < 0.0001 | |  |
| **pwC compound2190** | t = 9.92, p < 0.0001 |  |  |  |
|  |  |  |  |  |
|  | alpha level: 0.005 |  |  |  |

| **test sample AOMIC** | |  |  |  |  |
| --- | --- | --- | --- | --- | --- |
|  | **pwC compound854** | **pwC compound2190** | **pwC 1000Brains** | **pwC eNKI** | **pwC GSP** |
| **pwC HCP** | t = -22.54, p < 0.0001 | t = -31.98, p < 0.0001 | t = -4.63, p < 0.0001 | t = -13.24, p < 0.0001 | t = -30.53, p < 0.0001 |
| **pwC GSP** | t = 5.77, p < 0.0001 | t = -3.90, p < 0.0001 | t = 24.12, p < 0.0001 | t = 17.38, p < 0.0001 |  |
| **pwC eNKI** | t = -10.40, p < 0.0001 | t = -19.97, p < 0.0001 | t = 7.76, p < 0.0001 | |  |
| **pwC 1000Brains** | t = -17.08, p < 0.0001 | t = -26.15, p < 0.0001 | |  |  |
| **pwC compound2190** | t = 9.09, p < 0.0001 |  |  |  |  |
|  |  |  |  |  |  |
|  | alpha level: 0.003 |  |  |  |  |
